# Will restricting the age of access to social media reduce mental illness in Australian youth?

**DOI:** 10.1177/00048674241308692

**Published:** 2024-12-30

**Authors:** Julie A Blake, Andre Sourander, Akina Kato, James G Scott

**Affiliations:** 1 Child Health Research Centre, The University of Queensland, South Brisbane, QLD, Australia; 2 Queensland Centre for Mental Health Research, The Park Centre for Mental Health, Wacol, QLD, Australia; 3 Child and Youth Mental Health Service, Children’s Health Queensland, South Brisbane, QLD, Australia; 4 Department of Child Psychiatry, University of Turku, Turku University Hospital, Turku, Finland; 5 INVEST Research Flagship, University of Turku, Turku, Finland

**Keywords:** Anxiety, depression, measurement, adolescence, social networking

## Abstract

In recent years, there has been a substantial increase in the prevalence of youth mental illness which has coincided with the growing use of social media throughout society. Studies have demonstrated associations between social media use by young people and mental ill health leading the Australian government to declare a ban on social media by Australians below 16 years of age. This paper aims to critically examine the existing literature reporting these associations and highlights key methodological limitations. We argue that before implementing any restrictive measures that will affect all young people across Australia, it is crucial to consider the evidence to ensure that the proposed legislation is well informed and evidence-based. We suggest there is inadequate evidence at this time to conclude that the rise in youth mental illness is attributable to social media. While the concerns about social media’s impact on youth mental health warrants attention, it is essential to consider alternative explanations and to conduct studies that inform highly impactful public health measures. Preventative strategies to address mental illness in young people must be feasible to implement, effective and not give rise to unintended harms.

## Introduction

There has been extensive commentary on the harms caused by social media to the mental health of young people. Recently, this has culminated in calls to ban social media access to Australians below 16 years of age, resulting in the Federal Government legislating to enforce minimum age restrictions for social media use (Prime Minister of Australia, 2024). While at first glance this may appear an appealing solution, present research does not yet provide strong theoretical grounds to justify the development and implementation of effective social media ban. The past decade has seen a significant transformation in social media usage, with a plethora of platforms now widely adopted among young Australians (Esteban, 2019). Today’s adolescents are the first generation to grow up immersed in social media from an early age, coinciding with a notable increase in mental health problems compared with previous generations (Australian Institute of Health and Welfare [AIHW], 2024). The correlation between the rising prevalence of youth mental illness and increased social media usage underscores the need to scientifically evaluate the impact of social media on youth mental health. However, current research methodologies that have measured the association between mental illness and screen time and social media use do not accurately capture the nuances of social media engagement. We aim to provide an overview of youth social media use, the mental health of young Australians, and the current evidence regarding their association. We outline methodologies required for future research to effectively advance the current understandings of the mental health impact of social media on youth.

## Social media use among young Australians

Government and academic reports provide insights into social media engagement trends among Australian youth. For instance, reports by the Office of the eSafety Commissioner (2018) and University of Sydney (Humphry et al., 2023) highlight the prevalent engagement among Australian adolescents with platforms, such as YouTube, Instagram, TikTok and Snapchat. Notably, the eSafety Commissioner’s *State of Play* report found around one-quarter of children (8- to 12-year olds) are already accessing social networking platforms, while Fardouly et al. (2020) showed that two-thirds of Australian children have at least one social media account by the time they reach their final primary education year. By 16 years of age, the majority of Australians have been using social media for a significant proportion of their young lives.

## The mental health of Australian youth

There has been a notable increase in the prevalence of mental health problems among Australian youth during the previous decade (Jorm and Kitchener, 2021; Shorey et al., 2022). The National Study of Mental Health and Wellbeing shows a 50% increase in the 12-month prevalence of mental disorders among Australians aged 16–24 years from 2007 to 2021, rising from 26% to 39% (ABS, 2022). Similar trends are observed in the NSW Population Health Survey, with 11% of 16- to 24-year-olds reporting high levels of psychological distress in 2013 compared with 28% in 2021 (Centre for Epidemiology and Evidence, 2021), and the Household, Income and Labour Dynamics in Australia survey which shows a rise in psychological distress from 18% in 2011 to 42% in 2021 (Wilkins et al., 2024). Importantly, some researchers suggest the increase in self-reported mental health symptoms may, in part, be a consequence of increased mental health awareness (Foulkes and Andrews, 2023). However, more objective data from mental health–related hospital presentations also demonstrate a rise in mental health problems in young people in recent years (Delaney et al., 2022).

The trend of rising mental health issues is not limited to Australia; it is reflected worldwide across various countries (McGorry et al., 2024). This increase has been notably worsened by the COVID-19 pandemic (Racine et al., 2021; Sicouri et al., 2023). Additional drivers of declining youth mental health include climate change (Crandon et al., 2022), rising costs of living (Mission Australia, 2024) and gender-based violence (Maidment et al., 2024). While it is inevitable that there are multiple factors which contribute to this trend, the influence of social media has been identified as a key contributor to the rise in mental illness in Australian youth (McGorry et al., 2023).

## Evidence of youth mental health harms from social media use

There has been a surge in studies examining the influence of social media use on youth mental health during the previous decade which have thus far yielded inconclusive and sometimes contradictory findings. Although recent reviews of the literature have found evidence of a positive relationship between social media use and worsened mental health, effect sizes are only small to moderate with authors cautioning against the conclusions of a causal relationship due to methodological limitations across studies. Three reviews reported a positive relationship between social media use, and anxiety and depressive symptoms (Fassi et al., 2023; Piteo and Ward, 2020; Ulvi et al., 2022). Another two reviews found evidence for a relationship between *problematic* social media use – characterised by addictive behaviour that negatively impacts mood and relationships – and depression (Huang, 2022) and social anxiety (Wu et al., 2024). The latter two reviews explored whether the social media measurement instrument used in each of the studies they reviewed moderated their results, with one (Huang, 2022) finding some social media use measures produced stronger effect sizes than others. Importantly, there is a lack of conceptual clarity and ongoing debate regarding the definition of ‘problematic’ social media use (Peng and Liao, 2023).

Meanwhile, there is mixed evidence as to whether social media use has been associated with self-injurious thoughts and behaviours, and suicidality. A meta-analysis by Nesi et al. (2021) found that social media use alone was not associated with self-injurious thoughts and behaviours; however, there was a moderate to large effect size found for the association between cyberbullying victimisation and self-injurious thoughts and behaviours. Another review found inconclusive evidence for a relationship between social media use and suicidal thoughts and behaviours, reporting that while the *frequency* of social media use tended to be positively associated with suicidal thoughts and behaviour, the cross-sectional nature of studies could not exclude reverse causation with young people turning to social media to manage suicidality (Macrynikola et al., 2021). Stronger associations are found for a relationship between social media use and outcomes related to body dysmorphia and disordered eating (Dane and Bhatia, 2023), although researchers highlighted that the links are largely indirect and mediated through social comparison. Other studies have also highlighted the adverse effects of social media on sleep quality in young people which partially mediates mental health outcomes (Alonzo et al., 2021; Yu et al., 2024).

Despite the accumulating evidence of harm to mental health, methodological limitations including reliance on cross-sectional data and heterogeneous measurement approaches of social media use impede the synthesis of findings and preclude definitive conclusions about causality (Orben, 2020; Valkenburg et al., 2022). In addition, an illness-focused approach to studying social media’s impact on mental health biases the reporting of associations (Sala et al., 2024). Social media can also promote wellbeing by fostering social connection, support, self-expression, and serving as a source of inspiration and learning (Shankleman et al., 2021). Notably, it can reach and support large numbers of young people, including those already facing mental health challenges, who may be harder to engage through traditional support channels (Robinson et al., 2016). Recognising both risks and benefits is critical to forming a more balanced perspective on social media’s role in young people’s mental health.

## Defining and measuring social media use

The evolving digital landscape presents considerable challenges in accurately assessing the impact of social media on youth mental health. Most studies rely on self-report data which are prone to biases and fail to capture the full spectrum of social media engagement. Mieczkowski et al. (2020) highlight the potential priming effects of survey design. They found that positive versus negative framing of questions relating to social media usage significantly influenced participants’ perceptions of their mental health symptoms, highlighting the need for careful consideration when designing questionnaires to reduce the risk of participant response bias.

Many studies fail to evaluate the quality of engagement with social media with most using simplistic measures which consider only duration or frequency of social media use (Bekalu et al., 2023). Moreover, Browne et al. (2021) stress a need for measures to assess both the purpose and productivity of social media use. They advocate for a comprehensive approach to measuring social media use that distinguishes active use from passive engagement and considers the balance of positive and negative experiences of social media. In support of this view, Roberts and David (2023) found that active social media engagement is associated with benefits, such as increased perceived social connection and wellbeing irrespective of the quantity of use. This nuanced understanding challenges the prevailing narrative of social media as solely detrimental to mental health and underscores the importance of considering positive aspects of online interactions. Alternative measurement methods such as technology-based tools (i.e., tracking apps) offer promise in capturing more objective data on social media usage patterns (Perez et al., 2023). Reliable collection of these data; however, requires further validation and consideration of contextual factors, particularly for younger age groups who may share devices with siblings and parents.

## Collecting the evidence to inform public policy

Given social media is now embedded in the lives of young people, banning its use by those under 16 years of age requires conclusive evidence of causative harm. Establishing if social media use is causative of mental illness in young people is arguably among the most important public health research needed in high-income countries. To gather this evidence, a nuanced understanding of social media use is essential. We propose the following methodological considerations to address this critical public health concern.

### Study design

Longitudinal studies are often argued for to establish causality. Studies that incorporate prospective follow-up, starting in pre-adolescence or earlier, would enable data collection on important transitions, such as the introduction of social media and acquisition of personal devices (Fardouly et al., 2020), which occur concurrently with the commencement of secondary education, puberty onset, and the emergence of mental health problems that most commonly occur in adolescents. The continued and rapid evolution of social media and the digital environment more broadly, however, may still render such studies inadequate to properly address this research question. Instead, researchers might consider adopting a time-trend approach whereby repeated cross-sectional surveys are administered using a similar methodology and target group to highlight the important temporal trends at the population level. Alternatively, multiple concurrent longitudinal studies of shorter duration with different ages at baseline (e.g. at 9, 11, 13 and 15 years) could accelerate the research findings.

### Social media measures

There is a need to identify valid measures of social media use that are less susceptible to bias. Exploration and validation of objective measures which use technology to continuously collect data would be preferable over self-report or single-item instruments.

### Consistent conceptualisation of social media use

Research must move beyond social media use as an exposure in the context of the duration and frequency of its use, and take a more nuanced approach to measuring *how* users are engaging with *(what)* social media platforms and *why*. The nature of user engagement with social media is diverse and driven in part by the characteristics of the app being used. The influence of social media on users can depend on the quality and depth of their engagement with the content and the balance of positive and negative experiences. For instance, it has been suggested that mental health and wellbeing outcomes differ for active vs passive social media use; that is, actively engaging through posting or commenting for example, compared with scrolling or consuming content without direct interaction. However, the utility of the passive–active framework is debated (Valkenburg, 2022), with negligible associations found between active and passive social media use and mental health and wellbeing. Further, subgroups of users who are marginalised or lack access to support derive some benefits but also experience increased anxiety from both passive and active use (Godard and Holtzman, 2024). These studies highlight dichotomous generalisations, such as social media being good or bad, or active use is helpful and passive use is harmful, are inaccurate. Most of the enormous research effort on social media and mental health to date does not offer helpful information regarding the methods by which social media use could be specifically targeted to effectively reduce youth mental health problems (Macrynikola et al., 2021).

### Inclusion of adequate confounding variables and examination of potential mediators and moderators

Adequate consideration of potential mediators, moderators and confounding factors that might influence the relationship between social media use and mental health must be incorporated into future research. Online experiences such as cyberbullying, cyber porn, cyber racism and discrimination, and other harmful interactions for example, are likely to negatively impact user wellbeing (Diomidous et al., 2016). User characteristics, including neurodiversity (Alfredsson Ågren et al., 2020; Hudson et al., 2023), familial and social relationship quality, sleep quality, levels of physical activity, socioeconomic status and parental mental health are also known influences of mental health problems in young people (Webb et al., 2022). Moreover, self-esteem may influence a young person’s vulnerability to the negative impacts of social media use through a greater propensity than others to self-evaluate through the means of social comparison and fear of missing out (Fardouly et al., 2020; Gori et al., 2023).

### Examination of digital parental monitoring

Parental monitoring has implications for how young people engage with the social media. Parental monitoring affects a young person’s vulnerability and level of exposure to negative online experiences. Parental support and engagement in navigating difficult online situations may also mediate the level of distress experienced by the young person. Parental oversight may also alter the type of social media apps a young person is permitted to use and type of engagement with those apps (e.g. what content can be accessed or posted). The frequency and duration of a young person’s social media use may be influenced by parental management of screen use (including through digital control apps, such as Family Link), where devices can be accessed (alone in bedroom vs family areas), and whether the young person is accessing social media through personal or shared devices. Research should also consider the consistency of rules for young people’s access (e.g. across households for children with more than one parental household) and consistency of parental monitoring patterns. For example, digital monitoring fatigue was commonly expressed by parent participants in the study by Humphry et al. (2023).

### Measurement of parental social media use

Beyond parental monitoring practices, parental social media use is an important but rarely addressed factor influencing the mental health of children. Parents who actively use social media may be better equipped to help their adolescent children navigate online spaces, encourage critical thinking about content and set healthy boundaries (Wallace, 2022). Conversely, excessive parental social media use can result in a reduction in parents’ emotional availability which negatively affects their children’s mental health and emotion regulation (Auxier et al., 2020; Beamish et al., 2018). Social media also provides parents with valuable resources to connect with others and access parenting information and support (Fierloos et al., 2022; Haslam et al., 2017), potentially enhancing their social support networks and benefitting their children indirectly. Notably, studies have found significant differences between mothers’ and fathers’ social media habits, including how they monitor and discuss its use with their adolescent children and its effects on the parent–child relationship (Wallace, 2022). Measuring parental social media use for *all* parents or primary caregivers is critical to understanding the causal pathways between technological changes and youth mental illness.

### Incorporation of qualitative research

Given the highly nuanced ways in which young people engage with social media, the inclusion of a qualitative research component in studies of social media and youth mental health can provide important contextual information to aid researchers in the interpretation of their findings and in drawing appropriate conclusions. Young people’s motivations for social media use are as diverse as they are. They encompass a range of purposes, such as social connection, entertainment, maintaining relationships with geographically distant family or friends, and accessing educational or personally relevant content (Humphry et al., 2023). Social media can also serve as a platform for seeking support during the times of psychological distress (Bailey et al., 2022; Popat and Tarrant, 2023).

### Nesting clinical trials

In drawing conclusions about the harms of social media use, it is important to consider whether modification of its usage results in meaningful clinical outcomes. Clinical trials where young people are randomly assigned to interventions to help them manage their online behaviours and examining the effect of this on their social media use and mental health is essential and should be part of any research effort to understand social media use and mental illness associations.

## Conclusion

There is converging evidence from research suggesting a possible causal association between social media use and mental health problems among young people. This has led to strident calls for interventions banning young people from accessing social media. We suggest three key problems with this proposal. First, it is not entirely clear that social media is responsible for the rising prevalence of mental health problems. It is plausible that social media use is harming youth mental health through other pathways, such as through parental social media use, which would not be addressed through a ban for young people. Second, the types of social media use that are harmful, and to whom they harm, are not well understood. A more nuanced understanding of how young people engage with social media is critical to effectively minimise unintended harms and while maximising benefits derived from restrictions. Third, it is not clear how a ban can be implemented and enforced. Experts warn that an age restriction is unlikely to work in the absence of appropriate age verification technology but caution that this would create new risks to individual’s privacy and cyber security (Manfield, 2024). Furthermore, a critical but missing element from the discussion is what platforms exactly will constitute social media? YouTube for example, is cited as the most frequently used social media platform in Australia, yet it also serves as a widely used tool for educational purposes which does not require engagement in a social media context (Moghavvemi et al., 2018). Furthermore, various strategies enabling young people to bypass age restrictions have already been identified, such as through the use of fake ID’s or virtual private networks (Humphry et al., 2024). It is unclear if and how, young people would be held accountable for breeching age restrictions and what (if any) consequences would be imposed for doing so. Finally but arguably most importantly, the voices of the young people being affected by the proposed ban are largely absent from these discussions (Humphry et al., 2024).

The forthcoming ban on social media will provide an opportunity to evaluate the real-world effect on restricting social media. It is critical that careful evaluation is undertaken to carefully examine both the benefits and harms of a social media ban on young people including those who are marginalised. We call for urgent investment in innovative high-quality pragmatic research which incorporates clinical trials to better understand and identify ways to prevent harm and manage social media. We also argue for investment in public health measures aimed at supporting parents and young people to practice healthier, safer social media use and to enhance family and community cohesion. Finally, we call on governments and other key stakeholders to negotiate more effectively with technology companies to develop solutions to improve the online safety of young people. The increase in youth mental illness is a complex problem unlikely to be solved by simple solutions or a single intervention. A public health approach delivering evidence-based preventative strategies in combination with effective accessible mental health services is needed to address mental illness in young Australians.
